# Examining the Moderating Role of the Intensity of Cigarette Smoking in the Relationship Between Job Demands and Burnout: A Cross-Sectional Study Among Japanese Workers

**DOI:** 10.7759/cureus.95857

**Published:** 2025-10-31

**Authors:** Hiroyuki Toyama, Jumpei Yajima, Suguru Iwano, Lauri Hietajärvi, Katja Upadyaya, Arnold Bakker, Katariina Salmela-Aro

**Affiliations:** 1 Department of Education, University of Helsinki, Helsinki, FIN; 2 Department of Human Studies, Beppu University, Beppu, JPN; 3 Psychology, Fukushima Medical University, Fukushima, JPN; 4 Work and Organizational Psychology, Erasmus University Rotterdam, Rotterdam, NLD

**Keywords:** burnout, japanese workers, job demands, smoking intensity, the job demands-resources model

## Abstract

Objective

A growing body of studies has shown that cigarette smoking is related to high levels of burnout. However, little is known about the role of smoking in the relationship between job demands and burnout. This study aims to address this gap by examining the moderating effect of smoking intensity (i.e., the average number of cigarettes smoked per day) on the relationship between job demands and burnout in Japanese workers.

Methods

A cross-sectional survey including questions about demographics, smoking status, job demands, and burnout was conducted among Japanese workers. Smoking intensity was measured using a single item referring to the average number of cigarettes smoked per day. Job demands were assessed using the New Brief Job Stress Questionnaire (for quantitative job overload) and the Quality of Work Index and Quality of Employment Index (for cognitive demands and emotional demands). Burnout was assessed using the 12-item version of the Burnout Assessment Tool. The data of 302 current smokers (70.2% male participants) were analyzed using a hierarchical multiple regression model. In Step 1, demographic factors such as age, sex, and employment status were entered. In Step 2 and Step 3, job demands and smoking intensity were added, respectively. Finally, in Step 4, the interaction term of job demands and smoking intensity was added to test whether smoking intensity conditions the job demands-burnout relationship.

Results

The result showed that the main effect of job demands on burnout was significant (Step 2: β=0.520, p<0.001), after controlling for the effect of demographic factors. This model accounted for 26.8% of the variance in burnout. In turn, the main effect of smoking intensity on burnout was also significant (Step 3: β=0.112, p=0.026). The inclusion of smoking intensity in the model resulted in accounting for an additional 1.2% of the variance in burnout. Furthermore, the effect of the interaction term of smoking intensity and job demands was also significant (Step 4: β=0.116, p=0.018). The final model accounted for an additional 1.3% of the variance in burnout. A post-hoc test revealed that the positive association between job demands and burnout was stronger for more intensive smokers (β=0.617, p<0.001, 95% CIs (0.486, 0.748)) than for less intensive smokers (β=0.406, p<0.001, 95% CIs (0.280, 0.532)).

Conclusion

The present results suggest that heavy smoking may undermine individuals' ability to effectively manage job demands, thereby increasing their susceptibility to burnout. Thus, although smoking offers temporary stress relief, heavy reliance on it may have a negative impact on occupational well-being. Accordingly, interventions aimed at reducing the burnout risks among smokers may benefit from incorporating smoking‐reduction or cessation strategies, alongside stress management tactics.

## Introduction

Cigarette smoking remains a major public health concern on a global scale [1]. In Japan, the Health Promotion Act was enacted in 2003, contributing to a steady decline in smoking rates [2]. Despite this progress, the health impacts of smoking remain significant and cannot be overlooked. According to a recent survey, smoking was the second-highest factor driving the most deaths and disability combined among Japanese people, estimated at approximately 142,800 deaths, and accounting for 23.7% of total deaths [3]. The leading cause of death attributed to smoking was lung cancer, with an estimated 51,000 deaths, corresponding to 78.9% of all lung cancer fatalities, followed by chronic obstructive pulmonary disease or COPD (approximately 12,300 deaths, accounting for 62.9% of all COPD-related deaths), and ischemic heart disease (approximately 15,900 deaths, representing 14.9% of all deaths from that condition) [3].

Smokers tend to use smoking as a means of coping with stress in daily life [4]. Due to their principal component, nicotine, which is a psychoactive substance, cigarettes produce pleasurable feelings immediately after use [5,6]. However, this effect is generally temporary, and it may exacerbate stress over time [6], ultimately leading to health problems. Research has shown that smoking is related to a wide range of health problems, including cardiovascular disease [7], cancer [8], and mental illness [9].

Among workers, burnout has been recognized as a significant correlate of smoking [10,11]. Specifically, burnout is a manifestation of chronic job stress characterized by four core symptoms: exhaustion, mental distance, cognitive impairment, and emotional impairment [12]. Exhaustion refers to feelings of depletion and a lack of physical and mental energy for work-related activities [12]. Mental distance involves feelings of detachment from an individual’s work or colleagues [12]. Cognitive impairment involves a decline in the regulation of cognitive processes such as memory, attention, and concentration [12]. Emotional impairment relates to a diminished capacity to regulate one’s emotions, such as sadness and anger [12].

The Job Demands-Resources (JD-R) model [13] provides a theoretical foundation to analyze burnout. This model proposes that workers’ well-being is determined by two fundamental job characteristics: job demands and job resources. Job demands are aspects of the job that require sustained mental, emotional, and/or physical effort of individuals, and hence come with psychological and/or physical costs, while job resources are those that serve to reduce job demands, achieve work goals, and foster personal growth [13]. According to this model, job demands trigger the health impairment process, wherein excessively high job demands erode individuals’ finite resources, ultimately leading to burnout [13]. Therefore, burnout is an outcome of high job demands. Supporting this notion, a meta-analysis has shown that job demands positively predict burnout [14].

The present study

A growing body of research has shown that smoking is positively associated with burnout [11,12], indicating that smoking may be a maladaptive coping mechanism in response to work-related stressors. Despite this potential, the impact of smoking on the relationship between job demands and burnout remained unexplored. Addressing this gap is crucial for at least two reasons. First, it offers an opportunity to refine and extend the JD-R framework by integrating health-risk behaviors (i.e., substance use) as potential moderators in the stressor-strain relationship. Second, such insights can inform the design of targeted workplace health interventions, particularly those aimed at reducing burnout risk among smokers.

As such, the present study aimed to examine the moderating effect of smoking (smoking intensity defined as the number of cigarettes an individual smokes daily) on the relationship between job demands and burnout. Drawing on the Conservation of Resources (COR) theory [15], we argue that the positive relationship between job demands and burnout is stronger for more intensive smokers compared to less intensive ones. COR theory states that stress arises from the loss, threat of loss, or insufficient gain of resources that individuals deem valuable [15]. Intensive smoking may act as a stressor because it encourages the loss of personal resources, such as self-control [16], health [17], and well-being [18]. Consequently, intensive smokers may be less capable of effectively coping with demands at work and thereby more prone to burnout. Thus, we proposed the following hypothesis: Smoking intensity moderates the relationship between job demands and burnout. Specifically, the positive association between job demands and burnout should be stronger for more intensive smokers than for their less intensive counterparts.

## Materials and methods

Participants

A cross-sectional survey was conducted in November 2023 among Japanese workers through a local survey agent (Cross Marketing, Tokyo, Japan). Participants were recruited from the company’s survey panel using a disproportionate stratified sampling method. Strata were defined by sex (male and female participants) and age group (20-29, 30-39, 40-49, 50-59, and ≥60 years), resulting in ten strata. Then, we distributed an email invitation containing a link to the online survey, along with information about the study’s main objectives, methodology, and ethical policies, to eligible monitors within each stratum. Based on the company’s historical response rate data, a total of 35,652 individuals were invited to achieve the target sample size of 1,500 participants. Participants were asked to carefully review the study description and complete the questionnaire within one week. The study protocol was approved by the Ethics Committee of Beppu University (approval number: 2023-4). Participation was voluntary, and participants could withdraw at any time without explanation or facing a disadvantage. Consent was confirmed upon completion of the questionnaire.

We obtained data from 1,540 individuals (response rate=23.2%). For the current study, we included only current smokers (n=1237), while individuals who did not complete their response regarding all study variables were excluded (n=1). Consequently, the data from 302 workers (70.2% male participants) were used for statistical analysis. This final data did not have any missing values.

Table 1 presents the demographic information of participants.

**Table 1:** Demographic profile of the participants (n=302)

Variable	Category	Proportion (%)
Age	20-29	10.3
	30-39	15.5
	40-49	17.7
	50-59	32.0
	60 and over	24.4
Sex	Male	70.2
	Female	29.8
Educational background	Junior high school or less	3.0
	High school or vocational school	33.8
	Junior or technical college	12.3
	Bachelor’s degree	48.0
	Graduate school	3.0
Occupations	Clerks	25.8
	Professional or technical	15.6
	Management	10.3
	Sales and marketing	16.2
	Service	9.9
	Production skills and operations	7.9
	Security	0.7
	Agriculture, forestry, and fishing	0.3
	Transportation and communication	4.3
	Other	8.9
Employment status	Permanent	84.4
	Non-permanent	15.6

Participants’ average age was 49.2 years (SD=12.4) with maximum participants in the 50-59 years age group (32.0%). The most frequent educational background of the participants was a bachelor’s degree (48.0%), followed by high school or vocational school (33.8%), and junior or technical college (12.3%). Nearly 67.9% of the participants belonged to the following occupations: 25.8% clerks, 15.6% professional or technical, 10.3% management, and 16.2% sales and marketing. Overall, 84.4% of the participants were permanent workers.

Measures

Smoking intensity was defined as the average number of cigarettes smoked per day. The raw index was categorized into five levels: 1 = 1-5 cigarettes, 2 = 6-10 cigarettes, 3 = 11-15 cigarettes, 4 = 16-20 cigarettes, 5 = 21 or more cigarettes. The distribution was 13.6% for the first level, 19.5% for the second, 25.2% for the third, 26.8% for the fourth, and 14.9% for the fifth. In the analysis, this variable was treated as a numeric variable.

Job demands were measured utilizing three items assessing quantitative job overload in The New Brief Job Stress Questionnaire [19] and two items assessing cognitive and emotional demands in The Quality of Work Index and the Quality of Employment Index [20]. Example items are “I have an extremely large amount of work to do” for quantitative job overload, “To what extent does your work demand concentration?” for cognitive demands, and “How often does your work require you to control your feelings?” for emotional demands. Prior to the survey, all items were reviewed by three experts in occupational psychology to ensure content validity and clarity.

Items for the quantitative job overload were scored on a four-point Likert scale ranging from 1 (not at all) to 4 (very much). Cognitive demands were scored on a five-point scale ranging from 1 (to a very low extent) to 5 (to a very large extent), whereas emotional demands were scored on a five-point scale ranging from 1 (never) to 5 (almost always). Confirmatory factor analysis (CFA) demonstrated a good fit of the three-factor model (χ² = 16.367 (df=11, p<0.001)), Comparative Fit Index (CFI)=0.993, Tucker-Lewis Index (TLI)=0.987, Root Mean Square Error of Approximation (RMSEA)=0.040, Standardized Root Mean Square Residual (SRMR)=0.022). In the analysis, we used the global mean of these demands as the score of job demands. The higher the score, the higher the job demands. Cronbach’s α of the final scale was 0.81 (0.86 for quantitative job overload, 0.68 for cognitive demands, and 0.87 for emotional demands).

Burnout was assessed utilizing the 12-item version of the Burnout Assessment Tool [12,21,22]. This scale measures four core symptoms of burnout: exhaustion, mental distancing, cognitive impairment, and emotional impairment. Example items include “At work, I feel mentally exhausted” (exhaustion), “I am cynical about what my work means to others” (mental distance), “At work, I have trouble staying focused” (cognitive impairment), and “At work, I feel unable to control my emotions” (emotional impairment). Responses were recorded on a five-point scale ranging from 1 (never) to 5 (always). CFA demonstrated a good fit of the four-factor model (χ² = 109.650 (df=48, p<0.001), CFI=0.970, TLI 0.959, RMSEA=0.065, SRMR=0.027). In the analysis, the global mean of the scale was used for the score of the burnout. The higher the score, the higher the levels of burnout. The scale showed high internal consistency, with Cronbach’s α estimated at 0.96.

The complete list of items used in this study can be found in the Appendix.

Statistical analysis

Data analysis was conducted using IBM SPSS Statistics for Windows, Version 28 (Released 2021; IBM Corp., Armonk, New York, United States) and Mplus 8.0 (Muthén & Muthén (2017), LA,CA). First, descriptive statistics, including means and standard deviations (SD) of study variables and correlation coefficients between them, were calculated using SPSS. Next, to check the risk of common method bias, we performed Harman’s single-factor test using CFA in Mplus. Finally, we tested the hypotheses utilizing a hierarchical multiple regression analysis in SPSS. First, demographic factors were introduced as control variables, including age, sex, and employment status (Step 1). Sex was coded 0=male and 1=female, and employment status was coded 0=temporary and 1=permanent. Next, job demands (Step 2) and smoking intensity (Step 3) were included to examine their main effect. Finally, the interaction effect of job demands and smoking intensity was entered to test the hypothesis (Step 4). In the regression models, all the continuous variables were mean-centered. Multicollinearity was assessed utilizing a variance inflation factor (VIF) test. If the value was greater than 10, the risk of multicollinearity being present existed [23]. To evaluate the model’s explanatory power and relative influence of individual predictors, an adjusted R-squared (R^2^) metric was utilized to ascertain the incremental contribution provided by each variable set. The moderating effect of smoking intensity was examined by a simple slope test using the computational tool provided by Preacher et al. [24]. Low smoking intensity was determined as a score 1 SD below the mean, and high smoking intensity was determined as a score 1 SD above the mean.

In all analyses, statistical significance for estimates was set at p<0.05. The magnitude of effect sizes (standardized regression coefficients: β) was defined as small for values between 0.10 and 0.29, medium for values between 0.30 and 0.49, and large for values of 0.50 or greater [25].

## Results

Descriptive statistics

Table 2 presents the results of the preliminary analysis.

**Table 2:** Means (M), standard deviations (SD), and intercorrelations of the study variables (n=302) Sex (0=male, 1=female); employment status (0=temporary, 1=permanent); *p<0.05; **p<0.01; ***p<0.001 (two-tailed).

	Study variables	M	SD	1	2	3	4	5
1	Burnout	2.11	0.92	－	－	－	－	－
2	Job demands	2.54	0.71	0.519***	－	－	－	－
3	Smoking intensity	3.10	1.26	0.109*	0.041	－	－	－
4	Age	49.16	12.40	-0.105*	-0.068	0.106*	－	－
5	Sex	－	－	0.020	0.065	-0.143**	0.135*	－
6	Employment status	－	－	-0.088	0.043	0.092	-0.214***	-0.200***

Job demands were positively associated with burnout (r=0.519, p<0.001). Smoking intensity was positively associated with burnout (r=0.109, p=0.029) and age (r=0.106, p=0.033), and negatively associated with sex (r=-0.143, p=0.006). Age was positively associated with sex (r=0.135, p=0.010) and negatively associated with burnout (r=-0.105, p=0.042) and employment status (r=-0.214, p<0.001). Sex was negatively associated with employment status (r=-0.200, p<0.001).

Common method bias check

Before hypothesis testing, we examined the potential common method variance with Harman’s single-factor test using CFA [26]. As a result, the single-factor model, wherein all items of job demands, smoking intensity, and burnout were loaded onto a single latent factor, showed a poor fit to the data (χ² =1228.217 (df=52, p<0.001), CFI=0.697, TLI=0.659, RMSEA=0.153, SRMR=0.115). This result suggested that common method bias is unlikely to be a serious concern in this study.

Hypothesis testing

Next, we performed the hierarchical regression analysis to test our hypotheses. Table 3 summarizes the results of the analysis.

**Table 3:** Hierarchical multiple regression for burnout B: unstandardized coefficients; β: standardized coefficients; SE: standard deviation; VIF: variance inflation factor; *p< 0.05; **p<0.01; ***p<0.001.

Explanatory variables	Step 1				Step 2				Step 3				Step 4			
	B	β	SE	VIF	B	β	SE	VIF	B	β	SE	VIF	B	β	SE	VIF
Age	-0.140	-0.131*	0.063	1.058	-0.101	-0.095	0.054	1.063	-0.119	-0.111*	0.054	1.086	-0.104	-0.097	0.054	1.102
Sex	0.032	0.015	0.126	1.051	-0.060	-0.028	0.108	1.058	-0.025	-0.012	0.108	1.081	-0.030	-0.014	0.108	1.082
Employment status	-0.306	-0.113	0.161	1.082	-0.369	-0.136**	0.138	1.084	-0.397	-0.146**	0.137	1.092	-0.393	-0.145**	0.136	1.093
Job demands (JDs)	－	－	－	－	0.513	0.520***	0.048	1.012	0.507	0.514***	0.048	1.016	0.500	0.508***	0.048	1.019
Smoking intensity (SI)	－	－	－	－	－	－	－	－	0.112	0.112*	0.050	1.050	0.103	0.102	0.050	1.057
JDs × SI	－	－	－	－	－	－	－	－	－	－	－	－	0.108	0.116*	0.045	1.024
R^2^	0.024				0.291				0.303				0.316			
Adjusted. R^2^	0.014				0.282				0.292				0.302			
ΔR^2^	0.024				0.268				0.012				0.013			
ΔF	2.429				112.171***				5.031*				5.646*			

In the first model (Step 1), age showed a significant and negative effect on burnout (β=-0.131, SE=0.063, p=0.027, 95% CIs (-0.265, -0.016)), representing a small effect size. However, this baseline model failed to account for the variance in burnout (p=0.065).

The second model (Step 2) showed that job demands had a positive effect on burnout beyond the demographic factors (β=0.520, SE=0.048, p<0.001, 95% CIs (0.418, 0.608)), indicating a large effect size. This model explained 26.8% of the unique variance in burnout (p<0.001).

Regarding the third model (Step 3), smoking intensity had a positive effect on burnout beyond job demands (β=0.112, SE=0.050, p=0.026, 95% CIs (0.014, 0.211)), representing a small effect size. This model explained the additional 1.0% of the variance in burnout (p=0.026).

Finally, the fourth model (Step 4) showed that the interaction term between job demands and smoking intensity had a positive effect on burnout (β=0.116, SE=0.045, p=0.018, 95% CIs (0.019, 0.198)). This model accounted for an additional 1.0% of the variance in burnout (p=0.018). Although the effect size was relatively small, interaction effects in social science research are typically modest in magnitude yet theoretically meaningful [27].

For all regression models, VIF values (1.012-1.102) were below the criterion, confirming that there was no multicollinearity problem.

**Figure 1:**
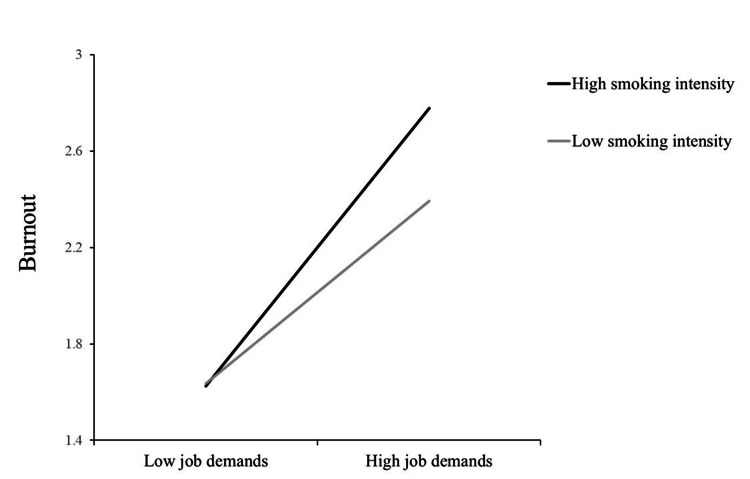
Moderating effects of smoking intensity

To investigate the moderating effect of smoking intensity, we conducted a post-hoc simple slope test. The result showed that the positive association between job demands and burnout was stronger for those with high smoking intensity (β=0.617, SE=0.067, p<0.001, 95% CIs (0.486, 0.748)) compared to those with low smoking intensity (β=0.406, SE=0.064, p<0.001, 95% CIs (0.280, 0.532)). Thus, our hypothesis was supported. Smoking intensity amplified the positive relationship between job demands and burnout. Figure 1 represents the moderating effect of smoking intensity.

## Discussion

This study aimed to examine the moderating effect of smoking intensity on the job demands-burnout relationship. To this end, we analyzed the cross-sectional data obtained from Japanese smokers using a hierarchical regression model. In what follows, we discuss the most important results of this study.

To begin with, individuals who experienced higher job demands reported higher burnout levels. This result is consistent with the JD-R model, which posits the positive association between job demands and burnout [14]. Job demands are characteristics of the job that necessitate an individual’s efforts to cope with them, thereby relating to psychological and physiological costs [14]. The JD-R model postulates that high job demands encourage the health impairment process, wherein individuals become more exhausted [14]. This theoretical assumption has also garnered support from recent meta-analyses, which showed that job demands positively predict burnout over time [15,16].

In addition, smoking intensity was positively associated with burnout, which highlights the harmful effects of intensive smoking. Importantly, smoking intensity explained the variance in burnout over and above job demands (defined as the global mean of quantitative job overload, emotional demands, and cognitive demands), suggesting that intensive smoking may be a salient risk factor for burnout, independent of major job-related stressors. Although smoking immediately produces euphoric sensations due to the effect of nicotine and may therefore be used to cope with stress [5,6], excessive smoking is likely to be counterproductive, leading to increased burnout. Yet, given that smoking temporarily reduces unpleasant feelings [5,6], it is also possible that the association between smoking intensity and burnout is bidirectional. That is, cigarette smoking may increase burnout, which in turn may intensify smoking, creating a vicious cycle wherein smoking and burnout mutually reinforce one another over time. This association should be investigated in future studies.

Moreover, the results showed a moderating effect of smoking intensity on the relationship between job demands and burnout. Specifically, the positive association between job demands and burnout was stronger for more intensive smokers, which fully supported our hypothesis. Thus, intensive smoking seems to magnify the detrimental impact of job demands on burnout. One possible explanation is that heavy smoking erodes individuals’ personal resources, which are crucial for coping with job demands. For example, intensive smoking can impair both mental and physical health [7-9,17,18], while withdrawal symptoms such as cravings can erode self-control resources [16]. Accordingly, individuals who smoke more intensively may have limited resources available to cope with job demands and may, therefore, be more susceptible to high levels of burnout. Our findings contribute to the JD-R research by shedding light on the role of smoking in the relationship between job demands and burnout. While the JD-R model has traditionally emphasized the interplay between job characteristics in explaining work-related well-being [13], relatively little attention has been paid to how employees’ health-related behaviors, particularly substance use, may influence these mechanisms. The present study filled this gap by examining the role of smoking intensity in the health impairment process among smokers and provided initial evidence that intensive smoking amplifies the positive association between job demands and burnout. Future research should verify the reproducibility of this finding and further investigate the role of smoking using more advanced methodological approaches.

From a practical standpoint, this finding highlights the importance of integrated interventions that address not only occupational stressors but also personal health behaviors. For employees who have an intensive smoking habit, reducing the number of cigarettes smoked may serve as an effective strategy to mitigate the detrimental effects of high job demands on well-being. Organizational practitioners should, therefore, incorporate smoking-reduction programs into their occupational health initiatives. For example, mindfulness-based programs to diminish stress reactivity and reduce substance use triggers [28] and cue-exposure therapy that systematically desensitizes individuals to smoking-related stimuli and builds coping skills [29] could be promising avenues to reduce the number of cigarettes they smoke. By combining workload management with tailored smoking-cessation resources, organizations may be able to address the burnout risk among smokers more effectively.

While this study provided innovative findings, it had the following limitations. First, as the data were collected through a web survey, the possibility of sampling bias could not be precluded. While this method has various substantial advantages (e.g., simplicity, low cost, and quick access to a large population), it may underrepresent specific individuals in the population, such as those with less access to the Internet [30]. Second, job demands were assessed using only selected items. Although the overall scale showed a sufficient internal consistency, the comprehensiveness of the construct measurement has been restricted. Moreover, smoking intensity (i.e., the number of cigarettes smoked per day) was categorized into five levels, which might have reduced variability and affected the precision of the estimates. Future research should adopt a comprehensive validated scale for assessing job demands and consider continuous measures of smoking behavior to improve measurement accuracy and analytic sensitivity. Third, this study utilized cross-sectional data, meaning that the results are correlational. To examine causal relationships between variables, longitudinal data that include at least two measurement points are needed. Fourth, the participants of this study were Japanese workers, thereby limiting the generalizability of the findings. Fifth, while this study focused on smoking intensity, it failed to distinguish between the cigarette subtypes that participants use (e.g., combustible cigarettes vs. e-cigarettes, containing nicotine vs. nicotine-free). Future work should incorporate cigarette characteristics alongside intensity to determine whether product type differentially moderates the job demands-burnout relationship. Finally, this study theorized smoking intensity as a moderator in the job demands-burnout relationship. However, given that smoking acts as a coping mechanism among smokers [4,6], it is also possible that smoking intensity plays a mediator role in this relationship. Thus, it would be interesting to investigate how job demands are related to burnout through smoking intensity over time.

## Conclusions

The present study offers an extension of the JD-R model by examining the role of smoking intensity as a boundary condition determining the relationship between job demands and burnout. We suggested that intensive smoking not only fails to buffer against work-related stressors but rather compromises employees’ capacity to meet demands in their work roles and thereby increases the burnout risk. The present findings underscore the maladaptive nature of cigarette smoking as a stress regulation strategy in occupational settings. Although it may offer temporary stress relief, heavy reliance on it seems to be rather counterproductive, increasing the vulnerability to burnout. Accordingly, intervention efforts aimed at reducing the burnout risks among smokers may benefit from incorporating smoking‐reduction or cessation tactics, alongside stress management strategies.

## Appendices

Instruments

**Table 4:** Instruments used in the present study

Scale	Subscale	Item
Smoking Intensity	－	On average, how many cigarettes do you smoke a day?
The New Brief Job Stress Questionnaire	Quantitative Job Overload	I have an extremely large amount of work to do.
		I can't complete work in the required time.
		I have to work as hard as I can.
	Cognitive Demands	To what extent does your work demand concentration?
		To what extent is your work intellectually challenging?
	Emotional Demands	How often does your work require you to control your feelings?
		How often does your work require you to hide your true feelings?
The 12-item version of The Burnout Assessment Tool	Exhaustion	I feel mentally exhausted.
		At the end of the day, I find it hard to recover my energy.
		I feel physically exhausted.
	Mental Distance	I struggle to find any enthusiasm for my work.
		I feel a strong aversion towards my job.
		I’m cynical about what my work means to others.
	Cognitive Impairment	I have trouble staying focused.
		I have trouble concentrating.
		I make mistakes because I have my mind on other things.
	Emotional Impairment	I feel unable to control my emotions.
		I do not recognize myself in the way I react emotionally.
		I may overreact unintentionally.
